# Portable Neuroimaging-Guided Noninvasive Brain Stimulation of the Cortico-Cerebello-Thalamo-Cortical Loop—Hypothesis and Theory in Cannabis Use Disorder

**DOI:** 10.3390/brainsci12040445

**Published:** 2022-03-26

**Authors:** Pushpinder Walia, Abhishek Ghosh, Shubhmohan Singh, Anirban Dutta

**Affiliations:** 1 Neuroengineering and Informatics for Rehabilitation Laboratory, University at Buffalo, Buffalo, NY 14228, USA; pwalia@buffalo.edu; 2 Postgraduate Institute of Medical Education & Research, Chandigarh 700020, India; ghoshabhishek12@gmail.com (A.G.); shubhmohan@gmail.com (S.S.)

**Keywords:** functional near-infrared spectroscopy, electroencephalogram, cortico-cerebello-thalamo-cortical loop, transcranial electrical stimulation, transcranial magnetic stimulation

## Abstract

Background: Maladaptive neuroplasticity-related learned response in substance use disorder (SUD) can be ameliorated using noninvasive brain stimulation (NIBS); however, inter-individual variability needs to be addressed for clinical translation. Objective: Our first objective was to develop a hypothesis for NIBS for learned response in SUD based on a competing neurobehavioral decision systems model. The next objective was to develop the theory by conducting a computational simulation of NIBS of the cortico-cerebello-thalamo-cortical (CCTC) loop in cannabis use disorder (CUD)-related dysfunctional “cue-reactivity”—a construct closely related to “craving”—that is a core symptom. Our third objective was to test the feasibility of a neuroimaging-guided rational NIBS approach in healthy humans. Methods: “Cue-reactivity” can be measured using behavioral paradigms and portable neuroimaging, including functional near-infrared spectroscopy (fNIRS) and electroencephalogram (EEG) metrics of sensorimotor gating. Therefore, we conducted a computational simulation of NIBS, including transcranial direct current stimulation (tDCS) and transcranial alternating current stimulation (tACS) of the cerebellar cortex and deep cerebellar nuclei (DCN) of the CCTC loop for its postulated effects on fNIRS and EEG metrics. We also developed a rational neuroimaging-guided NIBS approach for the cerebellar lobule (VII) and prefrontal cortex based on a healthy human study. Results: Simulation of cerebellar tDCS induced gamma oscillations in the cerebral cortex, while transcranial temporal interference stimulation induced a gamma-to-beta frequency shift. A preliminary healthy human study (N = 10) found that 2 mA cerebellar tDCS evoked similar oxyhemoglobin (HbO) response in the range of 5 × 10^−6^ M across the cerebellum and PFC brain regions (α = 0.01); however, infra-slow (0.01–0.10 Hz) prefrontal cortex HbO-driven phase–amplitude-coupled (PAC; 4 Hz, ±2 mA (max)) cerebellar tACS evoked HbO levels in the range of 10^−7^ M that were statistically different (α = 0.01) across these brain regions. Conclusion: Our healthy human study showed the feasibility of fNIRS of cerebellum and PFC and closed-loop fNIRS-driven ctACS at 4 Hz, which may facilitate cerebellar cognitive function via the frontoparietal network. Future work needs to combine fNIRS with EEG for multi-modal imaging for closed-loop NIBS during operant conditioning.

## 1. Introduction

In a neurobiological framework, the transition from misusing addictive drugs to substance use disorder (SUD) is increasingly shown to be related to neuroplastic changes in the structures and functions that promote and sustain SUD, including addiction—the most severe form of SUD [1]. The onset, development, and maintenance of SUD shows dysfunction in three main areas of the brain: the basal ganglia, the extended amygdala, and the prefrontal cortex [1]. Brain dysfunction can trigger different behavioral aspects of SUD, including substance-seeking triggered by substance-associated cues, reduced sensitivity to reward and heightened activation of brain stress systems, and reduced executive control. Adolescence is a critical “at-risk period” for all addictive drugs, including alcohol and cannabis, during which neuroplastic changes due to a less potent drug may facilitate substance-seeking of a more potent addictive drug. The differential nature of the interactions that occur between substance use and brain structure maturation across adolescence and into young adulthood has been highlighted in a recent work [2].

Cannabis is the most widely cultivated, trafficked, and abused illicit [3]. In 2018, an estimated 192 million people aged 15–64 used cannabis for nonmedical purposes globally [4]. The Global Burden of Diseases, Injuries, and Risk Factors Study (GBD) 2016 estimated that, across the globe, there were more than 22.1 million people with cannabis dependence [5]. Moreover, the same study calculated that cannabis dependence could account for 646,000 disability-adjusted life years globally. Significantly, cannabis dependence mainly affects young adults (20–24 years) and has a significant negative impact on these individuals’ growth and productivity and on the societies and nations to which they belong [4]. In addition to dependence syndrome, cannabis use is associated with an increased risk of psychosis [6], cognitive dysfunction, academic problems, and roadside accidents [7]. A review showed a consistent association between cannabis use and lower educational attainment and increased reported use of other illicit drugs [8]. In the United States, cannabis use disorder (CUD) is an escalating problem in young adults due to legalization [9], the National Survey on Drug Use and Health having reported an increased prevalence from 5.1% in 2015 to 5.9% in 2018 in 18–25-year-olds [10].

The psychoactive effects are primarily due to the type 1 cannabinoid receptor (CB1), the cannabinoid binding protein that is highly expressed in the cerebellar cortex [11]. CB1 is primarily found in the molecular layer of the most abundant synapse type in the cerebellum [11] that can shape the spike activity of cerebellar Purkinje cells [12]. Moreover, granule cell-to-Purkinje cell synaptic transmission can trigger endocannabinoid release [13], which may be important for information processing by cerebellar molecular layer interneurons [14]. This suggests that endocannabinoids could be essential to neurocognitive aspects of cerebellar function [11,13,15], and CB1 receptor downregulation in long-term chronic cannabis use may promote CUD [16]. Accumulating evidence also suggests cerebellar modulation of reward circuitry and social behavior via direct cerebellar innervation of the ventral tegmental area (VTA), including dopamine cell bodies (A1) in the VTA [17]. VTA dopamine (DA) signaling in the nucleus accumbens (NAc) and the medial prefrontal cortex (MPFC) [18] plays a crucial role in motivated behavior and cognition. Cerebellar neuropathological changes can result in aberrant dopaminergic activity in the NAc and MPFC [18,19], leading to dysfunctional behavior and cognition. Here, CUD-related cerebellar dysfunction is postulated to have a role in an aberrant dopaminergic activity that can include reward-related behaviors, information processing, and cognitive control [11,13,15]. In this hypothesis and theory article based on prior methodological developments [20], we present an application of portable neuroimaging-guided noninvasive brain stimulation of the cortico-cerebello-thalamo-cortical loop in CUD.

## 2. Hypothesis 1: Cerebellar Brain Inhibition in a Competing Neurobehavioral Decision Systems Model

**Figure 1:**
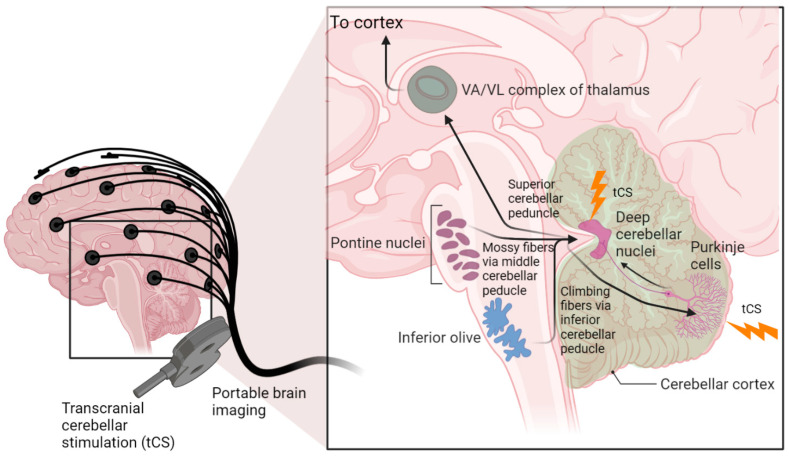
Cerebellocortical circuit. The cerebellum sends its output through the superior cerebellar peduncle, the contralateral red nucleus, and ventral anterior/ventral lateral nucleus of the thalamus to various cerebral areas, including the motor cortex, the prefrontal cortex, the parietal cortex, and the temporal cortex. Recent work has found that the frontoparietal network is disproportionately expanded in the cerebellum compared to the cortex. Transcranial cerebellar stimulation can affect the integration of sensory and cortical signals at the cerebellar cortex (Purkinje cells) as well as the deep cerebellar nuclei through which the cerebellum delivers its output to the cerebral cortex. (created using BioRender.com).

Research on repetitive transcranial magnetic stimulation (rTMS) for the treatment of substance dependence has shown encouraging results so far, especially concerning reducing drug cravings and improving cognitive outcomes [21,22,23]. However, NIBS’s effect is only transient and fades rapidly after treatment termination [23]. Craving is postulated as the failure of the normal inhibitory processes mediated by prefrontal cortex (PFC) regions to control reward processes mediated by the limbic system [24]. Although neuroimaging studies have implicated diverse PFC regions including dorsolateral prefrontal cortex [25], the right inferior frontal cortex has been implicated by human lesion mapping [26]. Therefore, excitatory rTMS to the executive control network [25] or inhibitory rTMS to the reward network can be postulated to result in decreased craving. Indeed, the left DLPFC is the most frequent anatomical target in clinical studies, followed by the right DLPFC [22]. Here, excitatory rTMS at the left DLPFC (not right DLPFC) has shown activation of the executive control network to reduce cravings in substance use disorders [27]. Figure 1 shows the cerebellocortical circuit for the competing neurobehavioral decision systems (CNDS) approach to planning NIBS intervention, which depends on the delineation of the functional organization of the prefrontal cortex [28] for portable neuroimaging-guided closed-loop NIBS [29]. Here, the activation of the executive control network via DLPFC is for the relative inhibition of the frontal–striatal circuits involved in limbic (amygdala, nucleus accumbens, ventral pallidum, and related structures) reward. In contrast, activation of the ventrolateral prefrontal cortex (VLPFC) can facilitate the cognitive control of attention and memory processing [30]—the ventral attention network. Here, the inferior frontal gyrus (IFG) in the VLPFC [31] is postulated to be crucial for memory retrieval (IFG pars orbitalis) [32] and post-retrieval control processes for amplifying inhibition downstream from the subthalamic nucleus [33] when substance-seeking is triggered by immediate attentional focus on substance-associated cues [34]. The dysfunctional response inhibition system for attentional focus on stimuli following substance-associated cues is postulated to trigger “automatic” goal-directed substance-seeking behavior where distinct neural circuits are responsible for the acquisition (during drug misuse) and “automatic” performance of the “learned” behavior (in SUD, addiction). Goal-directed behaviors are driven by brain structures, including the medial prefrontal and orbitofrontal cortices, hippocampus, and ventral and dorsomedial striatum, while sensorimotor cortices and the dorsolateral striatum mediate automatized/reflexive behavior. Within this brain network, the dorsomedial striatum (DMS) receives excitatory inputs from the PFC, whereas the dorsolateral striatum (DLS) primarily receives inputs from the sensorimotor and premotor cortices. In primates, the caudate nucleus and the putamen correspond to the DMS and DLS in rodents, where DLS has been shown to mediate stimulus–response habits [35]. This network mapping can be related to habitual performance, i.e., when the response is no longer flexible or adaptive [36]. Animal studies have shown distinct DMS and DLS activity patterns during the early acquisition stage that become similar during an automatized performance [36]. Extinction learning may enable learning of new contingencies via inhibition of the automatized response that will require facilitation of the inhibitory connections from the PFC to the subcortical regions to enable cognitive flexibility [37,38]. Here, a cortical–dorsomedial striatal circuit starting from the PFC is responsible for acquiring goal-directed actions, while a cortical–ventral striatal circuit mediates the performance [35]. Therefore, it is hypothesized that the response inhibition system can be facilitated by the activation of IFG for proactive control of attentional focus on stimuli [34,39] during cue-exposure therapy [40]. Then, a decrease in ventral striatum activity has been shown to correlate with treatment effects [41]. 

In this hypothesis and theory paper, we review cerebellar NIBS to reduce “craving”—a core symptom of any substance use disorder; however, defining “craving” is challenging [22]. Therefore, “cue-reactivity” is used as a closely related construct that can be measured using behavioral paradigms and imaging metrics (e.g., electroencephalogram, functional brain imaging, eye-tracking/pupilometry, heart rate) [22]. Besides the medial prefrontal cortex (MPFC) and cingulate cortex, which may predict relapse across multiple substances [22], we postulate that the cerebellum may also modulate the allocation of attentional resources [42] to cue stimuli relevant in “cue-reactivity.” Specifically, the default mode network, based in the ventromedial prefrontal cortex (vmPFC) and posterior cingulate cortex (PCC) [43], may directly modulate “cue-reactivity” in relapse for task-positive networks for substance-seeking. Whole-brain network studies show that the cerebellum and striatum are functionally connected with the cortical regions of the default mode network [44], which need further elucidation. Recent work has found that the frontoparietal network is disproportionately expanded in the cerebellum compared to the cortex [45], and a recent meta-analysis showed altered activation of the frontoparietal network, the ventral attention network, and the cerebellum during response inhibition tasks using non-addiction-related stimuli in adults with addiction [46]. Therefore, cerebellar NIBS may facilitate attentive executive function [42] in the Posnerian model to reduce “cue-reactivity.” Here, portable imaging metrics, including eye-tracking [47], electroencephalogram (EEG) [48], and functional near-infrared spectroscopy (fNIRS) [49], can provide insights into NIBS effects during a “cue-reactivity” test that is feasible in point-of-care settings than functional magnetic resonance imaging (fMRI) [50]. For example, EEG delta power has been postulated to be linked to increased activity of the dopaminergic brain reward system [51] and increased craving [52], so reduced EEG delta power can be related to therapeutic benefit. 

Multi-modal portable fNIRS–EEG joint imaging [53] is postulated to capture the subject-specific response for dosing NIBS. Here, inhibition of the reward network is postulated to be achieved by cerebellar rTMS [54] via cerebellar innervation of dopamine cell bodies in the VTA (Carta et al., 2019) [17]. Low-intensity rTMS is proposed to primarily affect the Purkinje cells in the cerebellum [55] via GABA-mediated inhibition of the deep cerebellar nuclei (DCN) in the fronto-cerebellar circuit [56]. Here, we augmented the CNDS theory [57,58] with recent evidence from neuroimaging studies of the fronto-cerebellar circuit, which interacts with the brain’s default mode network and is relevant in cognitive functions [19], and showed that cognitive control [59] may be diminished in the addicted brain along with memory, reward/saliency, and motivation/drive components [60]. Here, it may be possible to exert a longer-term effect via cerebellar NIBS because of its broader connections with the memory circuit and its role in habit formation [60]. Animal studies have shown a cerebellar contribution to extinction learning where the motor memory preserved in the cerebellum needs to be inhibited by the forebrain structures via the amygdala complex [61]. Therefore, neuroplastic changes in the cerebellum are postulated to be crucial for long-term therapeutic effects by reducing cerebellar “addiction” memory (lobule VIIb [60]). Indeed, a human study showed a detrimental impact of anodal cerebellar tDCS on the performance and timing of learned motor responses; however, extinction learning was not affected during the acquisition phase [62]. Here, cerebellar tDCS effects on motor learning can provide essential insights since motor symptoms can also be a characteristic of the disorder [63]. Based on these prior works, we suggest lobule VII (including Crus I, Crus II, and lobule VIIb)- specific cerebellar NIBS [64,65] to facilitate extinction learning toward substance-related cues in CUD. Here, cerebellar brain inhibition (CBI) is used in neurophysiological studies to characterize the inhibitory activity of the cerebellar cortex in the dentato-thalamo-cortical pathway [66]. Therefore, we first investigated the “knee” in the recruitment of the cerebellar primary motor cortex (M1) connection, or the CBI recruitment curve, at different intensities of the cerebellar TMS conditioning stimulus based on computational modeling and published experimental results [67].

## 3. Theory 1: Computational Modeling and Simulation of Cerebellar Brain Inhibition Measure 

**Figure 2:**
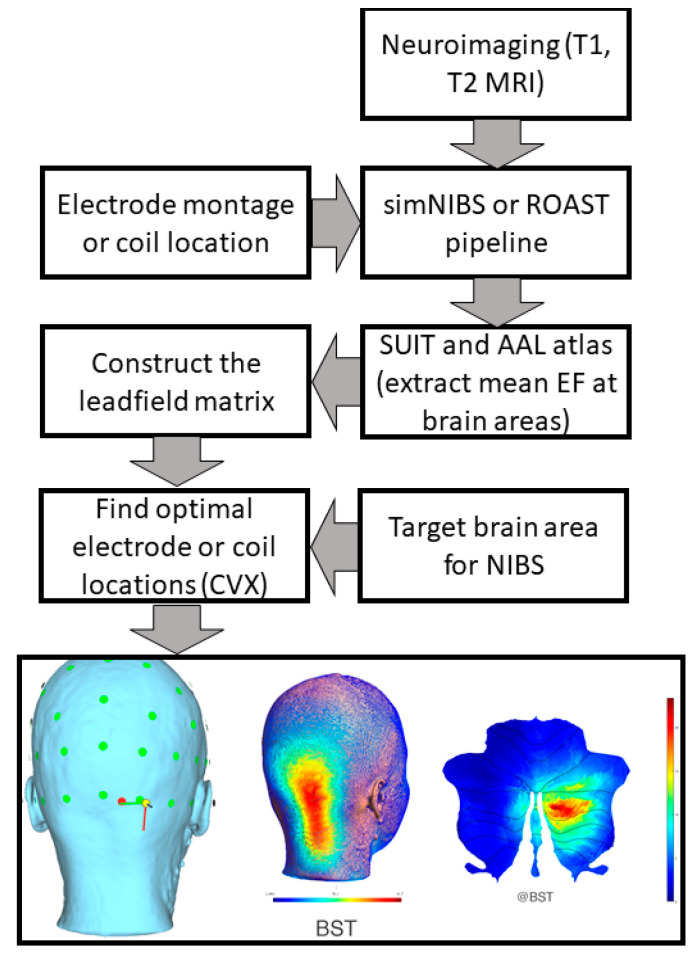
Computational pipeline for MRI-based optimization of non-invasive brain stimulation for a target electric field (EF) distribution using convex optimization (CVX). Bottom panel shows an illustrative example of TMS targeting Crus II at brainstem threshold (@BST).

The head model for computational modeling and simulation was created based on structural magnetic resonance images (MRI) from our prior work on the cerebellar lobule’s optimal stimulation (CLOS) pipeline (Rezaee and Dutta, 2019) [64]. Figure 2 shows the neuroimaging-guided NIBS pipeline using a subject-specific head model from the SPM12 segmentation algorithm (https://www.fil.ion.ucl.ac.uk/spm/software/spm12/ accessed on 30 December 2021) in MATLAB (Mathworks Inc., Portola Valley, CA, USA). The CLOS pipeline can use a realistic volumetric approach to simulate a transcranial electric stimulation (ROAST) pipeline [68] or SimNIBS [69] for finite element analysis of the electric field for electrode or coil optimization [64,70]. Lobule-specific cerebellar NIBS is crucial, since human functional neuroimaging has shown segregated fronto-cerebellar circuits [71], e.g., DLPFC-correlated activity was shown to span cerebellar Crus I/II lobules in its lateral and ventral extent. In contrast, MPFC-correlated activity spanned the cerebellar Crus I lobule. Here, Crus I preferentially correlated with MPFC, while Crus II preferentially correlated with DLPFC. Then, lobule-specific rTMS will require a neuroimaging-guided individualized approach for the delivery of cerebellar NIBS (details are provided in the Supplementary Materials based on our prior work [64]). Here, posterior cerebellar hemisphere structures, such as hemispheric lobule VI and Crus I, are significant in addiction [60] to be targeted with cerebellar NIBS [64].

**Figure 3:**
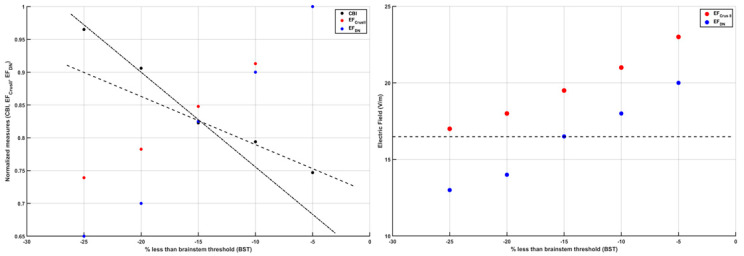
(**Left**) panel shows the change in cerebellar brain inhibition (CBI) from a neurophysiological study and change in normalized (by maximum) electric field strength at Crus II and dentate nuclei (DN) with the change in the intensity of the transcranial magnetic stimulation (TMS) as a percentage less than the brain stem threshold (BST). (**Right**) panel shows the electric field strength (V/m) at Crus II and dentate nuclei (DN) with the change in the intensity of the TMS as a percentage less than the BST. It is postulated that −15% less than the BST (dashed line in the right panel) is the TMS intensity at which the DN begin to be activated by the TMS (as represented by the change in the slope described by the dashed and dash–dot lines in the left panel).

The left panel of Figure 3 shows the CBI recruitment curve at different intensities of the cerebellar TMS conditioning stimulus based on our prior work [67]. The conditioning TMS intensity was reduced in 5% steps below the brainstem motor threshold (BST) up to −25%. BST was determined by corticospinal tract activation by single-pulse TMS with the double-cone coil placed over the inion. The left panel of Figure 3 also shows the computed mean electric field (EF) at Crus II and the dentate nucleus (DN), normalized by the maximum, at various conditioning TMS intensities (−5%, −10%, −15%, −20%, −25% BST). A “knee” was noticed around −15% BST when the CBI recruitment curve slope became flatter for further increase in the conditioning TMS intensity (i.e., a change point). This is postulated to be due to the stimulation of the DN (which is excitatory, shown by a blue marker in Figure 3) in addition to the Purkinje cells (which are inhibitory, shown by a red marker in Figure 3), resulting in a slower increase in CBI with increasing conditioning TMS intensity. The right panel shows the computed mean electric field (V/m) at Crus II and DN using CLOS [64], where the horizontal line denotes the DN mean electric field (V/m) at −15% BST, which is postulated to be the electric field (EF) threshold for DN activation. Here, all the mean EF (V/m) values at Crus II, which resulted in CBI (see left panel of Figure 3), were higher than the EF threshold for DN activation. 

Motor-evoked potentials (MEPs) cannot be generated at the non-motor areas, so lobule-specific cerebellar NIBS can be combined with portable fNIRS–EEG joint imaging [20,53,72,73] to identify individual NIBS dose–response relationships (as well as non-responders) (Rezaee et al., 2020b) [74]. In a feasibility study [74], we have shown that the combination of fNIRS and EEG would allow for noninvasive and simultaneous assessment of cerebral response to bilateral deep ctDCS of the dentate nucleus and cerebellar lobules VII–IX. Here, ctDCS was optimized for targeting the dentate nucleus [75] that stimulated the anterior and posterior lobes of the cerebellum, including cerebellar hemispheric lobules Crus I–Crus II and the dentate nucleus, which was postulated to modulate cerebrum activity in a different way to ctDCS of the posterior lobes of the cerebellum consisting of the hemispheric lobules VIIb–IX. The inset in Figure 1 shows the cerebellocortical circuit where cerebellar NIBS can be targeted not only at the cerebellar cortex (including Purkinje cells, which integrate sensory and cortical information [76] but also at the dentate nuclei, through which the cerebellum delivers its vast amount of output to the cerebral cortex [77].

## 4. Hypothesis 2: Cannabis Use-Related Dysrhythmia in the Cerebellocortical Circuit and Psychotic Disorder

**Figure 4:**
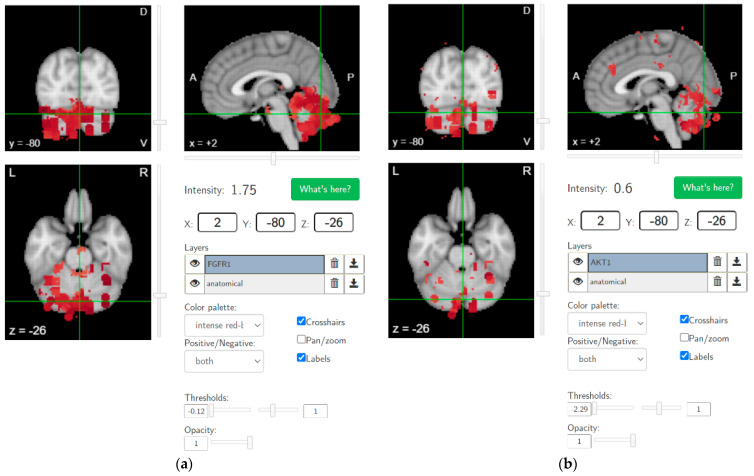
Brain-wide gene expression levels of (**a**) FGFR1 (thresholds: −0.12 and 1) and (**b**) AKT1 (thresholds: −2.29 and 1), as made available by the Allen Human Brain Atlas (from https://neurosynth.org/ accessed on 30 December 2021).

Recent studies have shown thalamocortical dysrhythmia in patients with schizophrenia spectrum disorder and individuals at high clinical risk for psychosis [78], which may be related to cannabis use in vulnerable individuals [79]. It is hypothesized that ameliorating maladaptive neuroplasticity in the cerebellum using NIBS will be crucial in CUD, since brain-wide AKT1 and FGFR1 gene expressions show hotspots in the cerebellum, as shown in Figure 4 (from https://neurosynth.org/ accessed on 30 December 2021), which makes it relevant for progression to psychotic disorder, especially with genetic predisposition [52]. In fact, the AKT1 genotype has been shown to influence the risk of psychosis, especially in young cannabis users [80]. Additionally, an altered function of fibroblast growth factor receptor (FGFR) signaling can be associated [81] where FGFR uses the endocannabinoid signaling system during neurodevelopment [82]. FGF7 and FGF22 have been shown to differentially promote the formation of inhibitory or excitatory presynaptic terminals [83] that may play a role in E/I balance [84]. FGFR1 possesses mechanisms to activate the AKT signaling pathway, which is relevant in the neurodevelopment of schizophrenia [85]. Protein kinase AKT1’s role in dopamine neurotransmission has been implicated in schizophrenia and psychosis [86]. FGF21 has been found to regulate sweet and alcohol preference correlated with reductions in dopamine concentrations in the nucleus accumbens, which coordinates reward behavior [87]. Interestingly, excitation/inhibition (E/I) balance is disrupted in schizophrenia [84], also based on a ‘phase zero’ brain organoid study [88], which can affect the neurodevelopment of the prefrontal cortex [89] (and cerebellum [84]), leading to propensity for substance abuse [90] in adolescence. In the cerebellum, the only output from the cerebellar cortex is represented by the inhibitory GABAergic Purkinje cells [91], while CB1 receptors are mainly expressed in the presynaptic terminals of granule cell molecular layer interneurons and climbing fibers that synapse onto Purkinje cells. CB1 receptor activity is required for long-term plasticity at parallel fiber–Purkinje cell synapses relevant for cerebellar learning. CB2 receptors in Purkinje cells may mainly participate in pathophysiological responses to exogenous cannabinoid compounds that can inhibit GABA receptor-mediated currents, potentially causing cerebellar dysfunction [92]. This will reduce the inhibitory tone in the cerebellum that can be investigated based on the effects on the primary motor cortex, i.e., CBI, which can be impaired in CUD [93] and schizophrenia [63].

Abnormal cerebellar volume also reflects genetic risk of addiction [60] where the E/I balance in the cerebellum during neurodevelopment may be facilitated with NIBS. Then, in CUD, it is postulated that PC modulation of DCN may get dysfunctional, which can be related to increased risk of psychosis and schizophrenia with familial/genetic risk factors [6,94,95])—a positive feedback cycle. For example, increased CB1 expression [11,13] in the molecular layer [11] can shape the spike activity of Purkinje cells [12]. Additionally, a decrease in Purkinje cell density [96] can lead to dysrhythmia in the cortico-cerebello-thalamo-cortical (CCTC) loop, and increasing residual Purkinje cell excitability with NIBS may ameliorate that dysrhythmia. Here, dysrhythmia in the CCTC loop as an extension to thalamocortical dysrhythmia [97] is postulated in CUD. In this hypothesis and theory paper, we further hypothesize that cerebellar NIBS can facilitate the amelioration of CUD-related dysrhythmia in the CCTC loop as an adjuvant treatment to operant conditioning (shown feasible in maladaptive motor control [98]) in a visual cue-reactivity paradigm using a virtual reality (VR) interface. Specifically, transcranial electrical stimulation (tES), a NIBS modality, is translatable to low-cost (<$150) mobile devices, allowing remote delivery of cerebellar NIBS in conjunction with VR-based cognitive operant conditioning in a low-resource home-based setting [99]. Therefore, we have established methods for portable neuroimaging-guided noninvasive brain stimulation that is presented for rational dosing of cerebellar NIBS [20] in CUD based on the insights gained from neuroimaging research on the cerebellum and addiction [60]. In this hypothesis and theory paper, we also investigated the transcranial temporal interference stimulation (tTIS) approach [100] using computational modeling and simulation of a CCTC loop model [101], which is presented next.

## 5. Theory 2: Computational Modeling and Simulation of tTIS-Based Amelioration of Dysrhythmia in the Cortico-Cerebello-Thalamo-Cortical Loop

**Figure 5:**
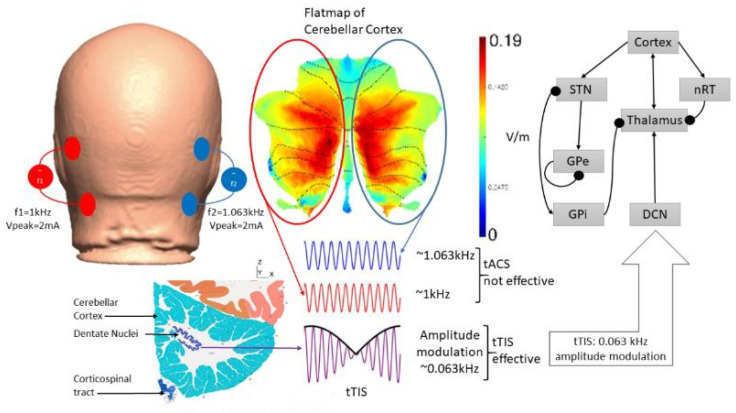
An illustration of the postulated transcranial temporal interference stimulation (tTIS) approach for deep cerebellar NIBS, where two tACS sources with frequencies f1 = 1 kHz and f2 = 1.063 kHz are combined for amplitude modulation at 0.063 kHz in the deep cerebellar nuclei (DCN) regions. The thalamocortical basal ganglia network with DCN from [104] is presented for tTIS modeling; arrows denote excitatory connections and round arrowheads denote inhibitory connections. http://atlas.brain-map.org/.

**Figure 6:**
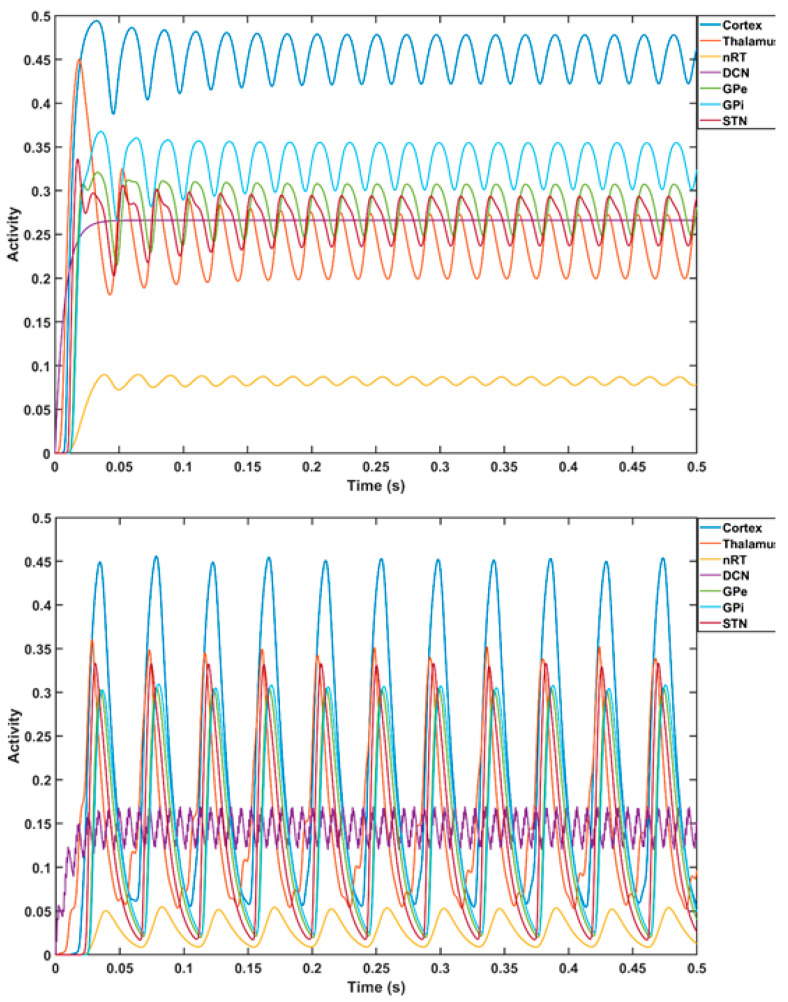
Computational modeling of thalamocortical basal ganglia with the cerebellum [104]. Top plot shows the cortical gamma frequency oscillations (in dark blue color) with a constant external input (transcranial direct current stimulation) to the deep cerebellar nuclei. The top plot also shows the oscillations in other components of thalamocortical basal ganglia model with the cerebellum—the excitatory ventralis intermedius (Vim) nucleus and the inhibitory reticular nucleus (nRT), an excitatory population of the deep cerebellar nuclei (DCN), an excitatory population representing the subthalamic nucleus (STN), and two inhibitory populations representing the external part of the globus pallidus (GPe) and the internal part of the globus pallidus (GPi). Bottom plot shows the transition of the cortical oscillations to beta frequency with tTIS of DCN at a 63 Hz.

Prior work [102] has identified a gamma-to-beta frequency shift as a marker of sensory gating that was found to be deficient in schizophrenia. Additionally, previous results have shown that gamma and beta frequency oscillations occur in the neocortex in response to sensory stimuli over various modalities [103]. Therefore, portable neuroimaging of the cerebellar tES response with combined fNIRS–EEG can guide tES dosing based on general linear modeling of dose–response relationships [74]. Here, we computationally investigated a tTIS approach [100] for cerebellar tES using a CCTC loop model [101] that took the average firing rates of the Purkinje cells (PCs) and deep cerebellar neurons (DCNs) to be 63 Hz and 56.6 Hz, respectively. For computational modeling of thalamocortical basal ganglia with the cerebellum [104], we selected f2–f1 = 63 Hz for the amplitude modulation of DCN by tTIS [105] (see Figure 5; further details are included in the Supplementary Materials). The thalamocortical basal ganglia model with the cerebellum [104] integrated two thalamic populations, the excitatory ventralis intermedius (Vim) nucleus and the inhibitory reticular nucleus (nRT), with an excitatory population of the deep cerebellar nuclei (DCN), an excitatory population representing the subthalamic nucleus (STN), and two inhibitory populations representing the external part of the globus pallidus (GPe) and the internal part of the globus pallidus (GPi), as shown in Figure 5. The model consisted of seven first-order coupled differential equations that simulated the gamma-band oscillations (>30 Hz) for a constant external input to the DCN (details in the Supplementary Materials). Here, a gamma-to-beta frequency shift can be considered a marker of sensory gating [103] that is postulated to be underpinned by cerebellum–hippocampal interactions [106,107]. Cerebellum–hippocampal connections have been found via the ventrolateral and laterodorsal thalamus in mice [108] and need further investigation in humans. However, for our computational simulation based on a published model [104], we postulated an effect of cerebellar tES on gamma-to-beta frequency shift as a marker of sensory gating triggered by substance-associated cues in VR, where interactions between sensory and motor cortices can be modulated by the cerebellum [109]. While ctDCS of the DCN induced gamma oscillations (top panel of Figure 6), the bottom panel of Figure 6 shows that tTIS of the DCN at 63 Hz amplitude modulation could lead to gamma-to-beta frequency shifts. Here, gamma frequency oscillations at the cortex can be generated with constant input (i.e., tDCS [74,75]) to the DCN, while tTIS of DCN at 63 Hz beats frequency (burst stimulation) led to beta frequency oscillations at the cortex (computational modeling details are included in the Supplementary Materials).

## 6. Hypothesis 3: Portable Neuroimaging-Guided NIBS to Reduce Inter-Individual Variability

Inter-individual differences in cerebellar NIBS effects on cerebrum activity are postulated to be measured by fNIRS–EEG joint imaging covering the prefrontal cortex, the primary motor cortex, and the supplementary motor area based on our prior work on healthy humans and stroke survivors [20]. Here, fMRI studies [71] have shown distinct PFC regions functionally connected to the multiple areas of the human cerebellum, e.g., Crus I with the MPFC, Crus II with the DLPFC. We propose a novel approach using latent variables from fNIRS and EEG [74] using a general linear model (GLM) [110] to study the effects of ctDCS. This was based on our prior work that showed that ctDCS electrode montages could be optimized to stimulate different parts or lobules of the cerebellum [64]. Specifically, we found [74] that bilateral ctDCS of combined anterior and posterior lobes of the cerebellum, including cerebellar hemispheric lobules Crus I–Crus II and the dentate nucleus, resulted in increased canonical scores of oxyhemoglobin (O2Hb) concentration changes as well as an increased canonical EEG score from the pre-ctDCS baseline at the contralateral (to the anode) PFC. In contrast, bilateral ctDCS of the hemispheric lobules VIIb–IX resulted in a small decrease in the canonical scores of O2Hb concentration changes and EEG from the pre-ctDCS baseline at the contralateral (to the anode) PFC from the pre-ctDCS baseline. Here, distinct areas of the PFC are functionally connected to lobule VII of the cerebellum [48], i.e., Crus I with the MPFC, Crus II with the DLPFC, ventral VIIB with the anterior prefrontal cortex (APFC). However, lesion heterogeneity led to inter-individual variability in the post-stroke fNIRS–EEG response [74], which accounted for the interindividual differences in ctDCS effects. Addressing heterogeneity is also important in CUD, since inter-individual genetic variations influence cerebellar volume [60] that affects electric field distribution [70]. 

## 7. Theory 3: Portable Neuroimaging-Guided Subject-Specific NIBS Application

**Figure 7:**
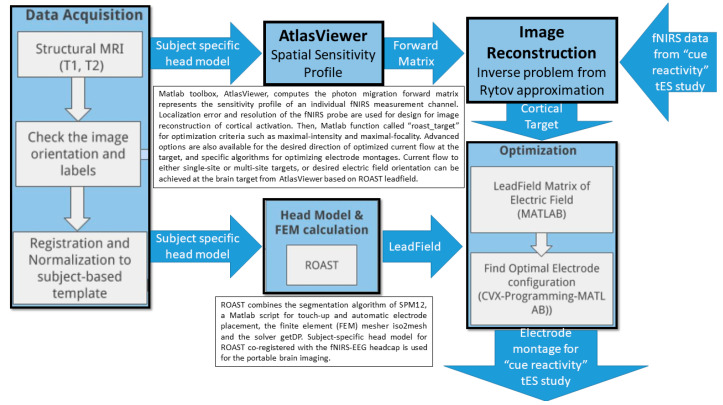
Computational pipeline for portable neuroimaging-guided transcranial electrical stimulation.

It is crucial to individualize NIBS treatment where an open-source realistic volumetric approach to simulate a transcranial electric stimulation (ROAST) pipeline [68] can provide the electrode montage with ‘maximal focality’ optimization criteria to target response inhibition brain activation with a 4 × 1 high-definition (HD) tDCS montage [111] (see Figure 7). In our prior work, we have optimized bipolar ctDCS montages for lower-limb motor representations and dentate nuclei in stroke survivors [75]. Here, motor representations are dual, whereas non-motor representations (attentional/executive and default-mode) are triadic in each cerebellar cortical hemisphere (lobules VI–Crus I; lobules Crus II–VIIB; lobules IX–X) [112]. Three functional domains were found in the cerebellar cortex, i.e., the functional gradients in the cerebellum, where the Crus I–II intersection is the intersection of the first and second default-mode representations [112]. Viral tracing studies in nonhuman primates have shown Crus I–II to have projections only to the prefrontal cortex [113]. Furthermore, functional MRI studies have shown Crus I connectivity with the MPFC and Crus II connectivity with the DLPFC [71]. Therefore, in this computational modeling and simulation study, we optimized bilateral electrode montage for non-motor representation in the cerebellar hemisphere, namely, the lobules VI–CrusI/II–VIIb, using a CLOS pipeline [64].

**Figure 8:**
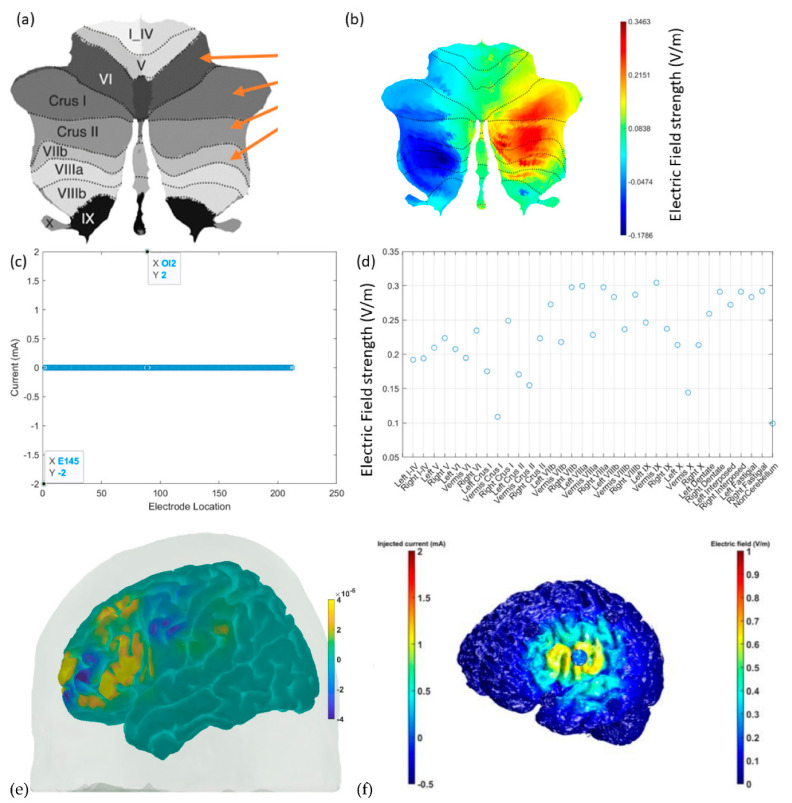
(**a**) A spatially unbiased atlas template (SUIT) of the human cerebellum and the ctDCS/ctACS targets shown with orange arrows for the cerebellar lobule’s optimal stimulation (CLOS). (**b**) The electric field (V/m) strength in SUIT results from CLOS. (**c**) Electrode location results from CLOS for 2 mA ctDCS/ctACS. (**d**) The cerebellar lobular and subsectional electric field (V/m) results from CLOS. (**e**) fNIRS HbO (in M) brain activation at the inferior frontal gyrus, which can be targeted with HD-tDCS. (**f**) HD-tDCS montage to facilitate downstream inhibition from the subthalamic nucleus when substance-associated cues trigger attention.

Figure 8 shows the results from CLOS optimization for maximum electric field strength at the non-motor representation, right lobules VI–CrusI/II–VIIb [64] based on a spatially unbiased atlas template (SUIT) of the human cerebellum [114] (see Figure 8a). Figure 8b shows that the optimized electric field strength in SUIT is focused (>0.2151 V/m) at the CrusI/II–VIIb lobules, where 2 mA at OI2 and −2 mA at E145 were found to be optimal (see Figure 8c). Then, optimized cerebellar lobular and subsectional electric field strength showed that the deep nuclei received comparable electric field strength to the cerebellar cortex (see Figure 8d). Figure 8e shows the feasibility of the fNIRS HbO-based brain activation measure at the VLPFC, including the inferior frontal gyrus [29], which was targeted with 4 × 1 HD-tDCS, as shown in Figure 8f, to facilitate ventral attentional control processes during VR-based extinction learning by amplifying downstream inhibition from the subthalamic nucleus for sensory gating [115].

## 8. Hypothesis 4: Portable Neuroimaging for Online Monitoring and Driving Cerebellar NIBS

Our computational modeling [116] showed the feasibility of evaluating the acute effects during the first 150 s of primary motor cortex tDCS in healthy humans using a fNIRS-based measure of blood volume. Additionally, in prior work [74], we found a linear relationship between electric field distribution and the HbO response using GLM analysis of variance (ANOVA). Therefore, we postulated that fNIRS of the cerebellum and the cerebrum can be used to monitor the effects of NIBS on the cortico-cerebello-thalamo-cortical loop. This was based on the SPM12 segmented head model and freely available Monte Carlo photon transport software (tMCimg) in the AtlasViewer [117] that were used to compute the fNIRS sensitivity profile [72]. Here, we aim for online monitoring and driving cerebellar NIBS to address dysfunctional sensory/sensorimotor gating, including prepulse inhibition, found to be deficient in cases of chronic cannabis use [118] and schizophrenia [119]. Our proposed fNIRS application for driving cerebellar NIBS was based on Marek et al. [45], who found that cerebellar blood oxygen level-dependent imaging signals temporally lag in the cortex, where infra-slow activity (0.01–0.10 Hz) and delta band (0.5–4 Hz) activity are propagated in opposite directions between the cerebellum and cerebral cortex. Therefore, tES with transcranial direct current stimulation (tDCS) and transcranial alternating current stimulation (tACS) were investigated for neuromodulation of the cerebellum and cerebral cortex to establish the following closed-loop NIBS theory.

## 9. Theory 4: fNIRS-Driven Cerebellar NIBS

**Figure 9:**
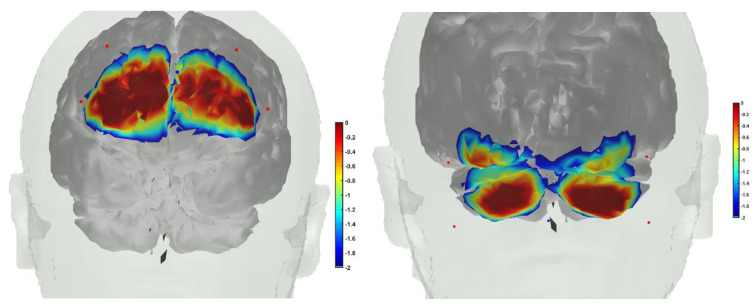
(**Left**) fNIRS sensitivity profile, where sources were positioned at AF7, AF3, AF8, AF4, and the detector was placed at the FPz. (**Right**) fNIRS sensitivity profile, where sources were positioned at PO7, PO9, PO8, and PO10, and the detector was placed at the Iz.

**Figure 10:**
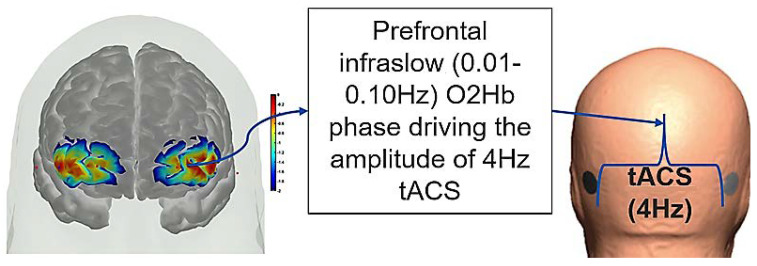
Illustration of the PFC phase–amplitude-coupled cerebellar tACS approach. Phase of the infra-slow (0.01–0.10 Hz) oxyhemoglobin (O2Hb) oscillations at the left PFC driving the amplitude of the 4 Hz cerebellar tACS optimized for targeting the lobules VI–CrusI/II–VIIb.

We conducted a feasibility test of fNIRS in young and healthy subjects to drive (phase–amplitude-coupled) cerebellar tACS (ctACS) at 4 Hz using endogenous infra-slow (0.01–0.10 Hz) PFC oxyhemoglobin concentration changes (HbO). Figure 9 shows the fNIRS sensitivity profile for the frontal cortex (left panel of Figure 9) and cerebellum (right panel of Figure 9) for a specific optode montage (confirmed with fOLD [120]. We found from our MRI-based head model in AtlasViewer that fNIRS sensitivity was mainly at Crus I–II of the cerebellum. Here, we postulated immediate NIBS effects on blood volume (measured by fNIRS) [116]. Furthermore, we postulated the feasibility of the prefrontal cortex (PFC) phase–amplitude-coupled closed-loop cerebellar tACS, as shown in Figure 10. Here, fNIRS-driven 4 Hz ctACS at ±2 mA (max) is expected to facilitate cerebellar brain inhibition [121] better than 2 mA cerebellar tDCS (ctDCS), that was evaluated based on fNIRS imaging [74].

Ten young and healthy right-handed subjects (8 males and 2 females, 21–25 years of age) volunteered for this study [122]. The session consisted of a block design of 2.5 min baseline, ctDCS/ctACS for a period of 5 min, and 2.5 min post-intervention measures. In this healthy human study, fNIRS was conducted using NIRSPORT 2 (NIRx Medical Technologies, Los Angeles, CA, USA). Our optode montage consisted of 12 long-separation (~3.5 cm) sources, 3 long-separation detectors (LD), and 3 short-separation (<1 cm) detectors (SD) that covered the PFC (4S, 1LD, 1SD), sensorimotor cortex (4S, 1LD, 1SD), and CER (4S, 1LD, 1SD). This long-separation optode montage was selected to match our low-channel count montage (Octamon+, Artinis Medical Systems, Netherlands) used in the stroke study where fNIRS sources were positioned at AF7, AF3, AF8, AF4, CP4, FC4, CP3, and FC3, and the two detectors were placed at the Cz and FPz with a source–detector distance of around 35 mm [74]. For CER fNIRS in this healthy human study, our fNIRS sources were positioned at PO7, PO9, PO8, and PO10, and the detector was placed at Iz, based on the fNIRS Optodes’ Location Decider (fOLD) [120]. Here, tES applied low currents around 2 mA that generated cortical electric fields less than 1 V/m [123], which has shown entrainment effects in the case of tACS [124]. We propose combined fNIRS and EEG to monitor and dose tES, including entrainment effects, based on prior works [53,72,73,74].

We used ctDCS/ctACS electrode montage for non-motor representation, i.e., lobules VI–CrusI/II–VIIb, using EEG locations [75]. The amplitude of the 4 Hz ctACS with optimized montage was driven by the phase of the infra-slow (0.01–0.10 Hz) HbO oscillations at the left PFC, found using the Hilbert transform for the analytic signal using a 60 s sliding window (see Figure 10). The maximum tACS amplitude was set at ±2 mA in the Starstim 8 tES device (Neuroelectrics). We compared ctACS and ctDCS effects based on fNIRS oxyhemoglobin concentration changes (HbO) at the prefrontal cortex (PFC) and cerebellum (CER). The session consisted of a block design of 3 min rest and a ctDCS/ctACS duration of 5 min, which was chosen based on prior works that showed significant increases in cortical excitability [125,126] and cerebral blood flow changes [127]. Our PFC optode montage covered MPFC and partly the DLPFC and VLPFC, as shown in the left panel of Figure 10. This is important, since the Crus I–II intersection is the intersection of the first and the second default-mode representations [112].

**Figure 11:**
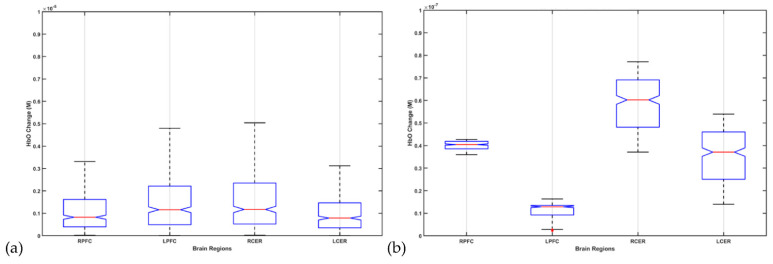
Box-plot of post-intervention HbO change from the pre-intervention baseline due to 2 mA ctDCS in (**a**) and ±2 mA (max) phase–amplitude-coupled ctACS in (**b**).

The fNIRS data processing was conducted using the open-source HOMER3 toolbox [128] in MATLAB (Mathworks Inc., USA). The raw optical intensity signal was first converted into optical density (function: hmrR_Intensity2OD), then motion artifact detection and correction were conducted using a hybrid method based on the spline interpolation method and Savitzky–Golay filtering (function: hmrR_MotionCorrectSplineSG) [129] using default parameters. Then, bandpass filtering was conducted (function: hmrR_BandpassFilt:Bandpass_Filter_OpticalDensity) within 0.01–0.1 Hz, followed by conversion to oxyhemoglobin (HbO) and deoxyhemoglobin (HHb) concentration (function: hmrR_OD2Conc). Finally, the hemodynamic response function (HRF) was computed using the general linear model (GLM) (function: hmrR_GLM_new) with short separation regression performed with the nearest short separation channel. GLM determined the HRF during the stimulation period from the resting state using ordinary least squares [130] with a consecutive sequence of Gaussian functions (stdev = 0.5, step = 0.5). Figure 11a shows the box-plot of post-intervention HbO change, where 2 mA ctDCS evoked similar HbO change across brain regions (α = 0.01). However, ±2 mA (max) phase–amplitude-coupled ctACS evoked HbO was lower but still statistically different (α = 0.01) across those brain regions, as shown in Figure 11b. Moreover, increasing the fNIRS-driven ctACS current to ±4 mA increased HbO response in the 10^−6^ M range, which may affect the deep cerebellar nuclei (DCN) due to higher electric field strength. The HbO responses are shown in the Supplementary Materials.

## 10. Discussion

Our hypothesis and theory paper has presented computational modeling and simulation results for portable neuroimaging-guided NIBS, including cerebellar tTIS in CUD. Computational modeling of the cerebrocerebellar connections of the afferent pathway (cerebello-thalamo-cortical) and the efferent pathway (cortico-ponto-cerebellar), as shown in Figure 1, is described in the Supplementary Materials. Here, the dysfunctional bidirectional interactions between DLPFC and the cerebellum can lead to dysrhythmia, affecting sensory gating which may be ameliorated by cerebellar tES, as shown by the simulation in Figure 6. Therefore, tTIS needs to be explored in future studies for specificity in targeting the cerebellar cortex versus the DCN (see Figure 1), where NIBS intervention can be important in the early stages of CUD which disrupts gamma band brain activity [131]. Here, reduced gamma waves in CUD is postulated to play a causal role in the development of psychosis [6] in certain genotypes with expression in the cerebellum, as shown in Figure 4. The neurobiological substrate can be ∆(9)-tetrahydrocannabinol (THC) [131], the main psychoactive constituent of cannabis, where chronic administration was found to produce significant reductions in prepulse inhibition (PPI) that resemble PPI patterns in schizophrenia [132]. However, cannabidiol in cannabis can have opposite effects on PPI [133], which may be related to the antagonist of the human CB2 receptor [134,135]). In cannabis use-related psychotic disorders, we postulate a role of dysrhythmia of the CCTC loop (as an extension of thalamocortical dysrhythmia [97]) in sensorimotor gating, including negative and positive symptoms due to dysfunction in the cerebellar cortex circuit. Here, we postulate that cerebellar NIBS may ameliorate maladaptive plasticity as an adjuvant treatment to cue-reactivity training, where cerebellar maladaptive plasticity may promote cannabis use-related psychotic disorders in vulnerable individuals [79].

A key feature of psychotic disorders is the involvement of subcortical dopaminergic dysfunction [136]. Here, fundamental invasive neuroimaging studies in animal models can confirm the change of the cerebellar brain connection using cerebellar TMS-evoked dose responses for the dopaminergic circuits based on a multi-modal approach [137,138,139,140,141,142] by incorporating extracellular electrophysiology and fast-scan cyclic voltammetry (FSCV) [143] (tip diameter, ~1μm). Simultaneous multi-modal monitoring would incorporate (i) a local view (<100 μm) of rapid changes in dopamine (DA) concentration (≤10 ms), which will exert rTMS effects on VTA DA regulation in the MPFC and nucleus accumbens (NAc) subregions, and (ii) simultaneous electrophysiological data at the the VTA, NAc, and MPFC over multiple spatial scales spanning individual neuronal spiking, population ensemble activity, and local field potential (LFP) oscillations [18]. However, TMS-based neuromodulation approaches are not amenable to home-based settings, so tES should be investigated as an adjuvant treatment, cerebellar tDCS of Purkinje cells and DCN having been shown to be feasible [75]. In addition, cerebellar tACS has been shown to be feasible in modulating motor behavior [144]; however, evidence for addiction medicine is limited [22]. Recently, tES for deep brain stimulation has been shown to be feasible using temporally interfering electric fields [105], so we performed a proof-of-concept computational simulation study (results presented in Figure 6). Furthermore, NIBS of VLPFC, including IFG (see Figure 8), can facilitate proactive attentional control [34,39] during cue-exposure therapy [40], which needs to be evaluated in a future clinical study. Our hypothesis and theory paper has presented experimental methodological approaches from prior works [20] for application in the study of CUD in order to investigate neuroimaging-guided tES that can ameliorate CUD-related maladaptive plasticity and related dysfunctional cortical inhibition [93]. Furthermore, NIBS of the cerebellum in conjunction with the VLPFC (including IFG) is proposed as an adjuvant treatment during cue-exposure therapy for operant conditioning that may ameliorate chemical dependency and habit formation [145].

In this hypothesis and theory paper, we have also presented feasibility testing of fNIRS of the cerebellum and PFC in healthy humans. We found HbO response to ctDCS and ctACS using an optimized montage (targeting lobules VI–CrusI/II–VIIb). Specifically, we found that 2 mA ctDCS evoked similar (α = 0.01) HbO responses across cerebellum and PFC brain regions that may be related to the modulation of Purkinje cells as well as deep cerebellar nuclei (see Figure 8d or Figure 11) [70]. Here, tDCS can have effects on different cell populations that together will generate the effect, which will be difficult to delineate [67] without computational modeling. Then, ctACS at the theta band frequency can increase the inhibitory tone that the cerebellum exerts over the cerebrum due to postulated selective recruitment of cerebellar granule cells and Golgi cells [121] which may have better specificity than tDCS. This modulation of the parallel fiber–Purkinje cell synapse is postulated to lead to the modulation of HbO activity at the PFC that can then drive the ctACS via a phase–amplitude coupling in our PFC phase–amplitude-coupled ctACS approach (see Figure 10). Here, adequate lag in the phase–amplitude coupling may be necessary for causal elucidation of the cerebellum and PFC effects from the HbO time series. However, we did not implement subject-specific lag for fNIRS-driven ctACS in this preliminary study. Nevertheless, we found HbO change at the left PFC to be lower than at the right PFC (see Figure 11b) during fNIRS-driven ctACS (with the phase of the infra-slow HbO oscillations at the left PFC) that also resulted in a higher HbO change in the right than in the left cerebellum that needs further investigation in conjunction with EEG measure of prefrontal gamma activity. One limitation of our preliminary healthy human study is the small sample size, which can undermine the generalizability of the outcomes.

## 11. Conclusions

In this hypothesis and theory paper, we developed a NIBS approach to ameliorate learned ‘habitual’ stimulus–response association in SUD based on a competing neurobehavioral decision systems model. Here, an adjunct treatment with NIBS along with VR-based operant conditioning, e.g., cue exposure therapy, can address the dysfunctional response inhibition system. Specifically, the ventrolateral corticolimbic pathways may be more relevant than fronto-parietal global attention network from PFC to the parietal lobule to control attentional focus on stimuli during cue exposure therapy that can be facilitated with IFG tDCS. Then, results from computational modeling highlighted the facilitatory effects of deep cerebellar tDCS on the cortical gamma frequency oscillations that may be reduced in individuals with early psychosis symptoms in CUD. Then, transcranial temporal interference stimulation of deep cerebellar nuclei at 63 Hz can facilitate gamma-to-beta frequency shift during VR-based operant conditioning where gamma-to-beta frequency shift is a marker of sensory gating which may be reduced in individuals with early psychosis symptoms in CUD. Here, positive modulation of the endogenous brain oscillations during VR-based operant conditioning can be facilitated with closed-loop NIBS. Our preliminary healthy human study showed the feasibility of fNIRS-driven ctACS where driving 4Hz ctACS with the phase of the infra-slow HbO oscillations at the left PFC resulted in an increased HbO change at the right PFC and cerebellum than the left PFC and cerebellum. 

## Data Availability

The preliminary data presented in this study are available on request from the corresponding author. The data are not publicly available due to the ongoing study but will be available in the future at https://www.brainrhythm.org/ (accessed on 30 December 2021).

## Supplementary Materials

The following supporting information can be downloaded at: https://www.mdpi.com/article/10.3390/brainsci12040445/s1. Figure S1: An illustrative picture of transcranial temporal interference stimulation (tTIS) approach where two tACS sources with frequencies fl = l kHz and f2 = 1.063 kHz are combined for amplitude modulation at 0.063 kHz at the deep cerebellar nuclei (DCN). Figure S2: Leadfield vector for PO9h at the 30 cerebellar lobules in the X, Y, and Z directions. Figure S3: Leadfield vector for POI Oh at the 30 cerebellar lobules in the X, Y, and Z directions. Figure S4: Leadfield vector for Exx8 at the 30 cerebellar lobules in the X, Y, and Z directions. Figure S5: Leadfield vector for Exx7 at the 30 cerebellar lobules in the X, Y, and Z directions.
