# Supplementary material for: Portable Neuroimaging-Guided Noninvasive Brain Stimulation of the Cortico-Cerebello-Thalamo-Cortical Loop—Hypothesis and Theory in Cannabis Use Disorder
Source: Brain Sci. 2022 Mar 26;12(4):445. doi: 10.3390/brainsci12040445 (PMC9027826; doi:10.3390/brainsci12040445)
Supplement: Supplementary file 1 [file brainsci-12-00445-s001.zip › brainsci-1557799-supplementary.pdf]

Supplementary Material

1 Supplementary Data: fNIRS-EEG guided tES approach based on published dataset

Published dataset is based on the research protocol was approved by the All India Institute of Medical Sciences, New Delhi, India Institutional Review Board (IEC-129/07.04.2017). This current study extends our prior work (Rezaee et al., 2021) on fNIRS-EEG joint imaging (Guhathakurta and Dutta, 2016) of ctDCS-modulated PFC responses (Rezaee et al., 2021) by investigating cortical activation using AtlasViewer toolbox (Aasted et al., 2015). This fNIRS-EEG joint imaging approach can also be used for functional neuroimaging of cue reactivity in CUD (Schacht et al., 2013). Since we did not have fNIRS-EEG joint imaging data of cue reactivity in CUD, so we illustrate our fNIRS-EEG joint imaging approach to response inhibition during a virtual reality (VR)-based Balance Training (VBaT) (Rezaee et al., 2021). Figure 4 shows the computational pipeline (Guhathakurta and Dutta, 2016) where AtlasViewer toolbox (Aasted et al., 2015) was used to conduct a Monte Carlo simulation that provided a forward matrix representing each channel's spatial sensitivity profile to cortical absorption changes, i.e., the fNIRS sensitivity profile. The forward matrix, F, transformed from the voxel space of localized changes, X, to the optode measurement space, Y, i.e., $Y = FX$. The X for image reconstruction can be obtained by solving the inverse problem (Rytov approximation (Devaney, 1981)) using AtlasViewer toolbox (Aasted et al., 2015) in Matlab (Mathworks, Inc., USA), i.e., $X = F^T(FF^T + \alpha I)^{-1}Y$, where I is the identity matrix and α is the regularization parameter (=0.01). Here, Y is the post-ctDCS change in the canonical scores of O2Hb concentration from pre-tDCS baseline. The Monte-Carlo simulation was set up with 100 million photons being injected by each source using the default optical parameters for each layer of the head model. Then, based on the PFC activation during response inhibition in VBaT, a fully automated pipeline – realistic volumetric-approach to simulate transcranial electric stimulation (ROAST) version 3 (Huang et al., 2017) – was used to find the tES electrode placement for targeting the brain activation found from image reconstruction using AtlasViewer toolbox (Aasted et al., 2015) in Matlab (Mathworks, Inc., USA). Different optimization criteria, including maximal-intensity and maximal-focality, can be used in the Matlab (Mathworks, Inc., USA) function called "roast_target" based on a lead-field matrix from an individualized (MRI-based) head model. In this study, we used maximal-focality optimization criteria to target the response inhibition brain activation with 4x1 high-definition (HD) tDCS montage (Mikkonen et al., 2020).

2 Supplementary Figures: neuroimaging guided NIBS in cannabis use disorder

CLOS pipeline uses structural magnetic resonance images (MRI) images (T1, T2) to create an anatomically accurate subject-specific head model that can be used for computing electric field distribution in the brain for a given electrode or coil location. CLOS pipeline uses freely available computational packages (simNIBS (Saturnino et al., 2018), ROAST (Huang et al., 2017)) along with freely available brain atlases (AAL (Rolls et al., 2020, 3), SUIT (Diedrichsen, 2006)) for a leadfield based approach to optimize (convex optimization (Boyd and Vandenberghe, 2004)) the electrodes or TMS coil location. The optimization is based on the quasi-uniform assumption that the local polarization effect is proportional to the local electric field strength (Bikson et al., 2013). If we consider a set of N bipolar electrodes or TMS coil locations, then the quasi-stationary Ohmic relation from the stimulation array, s, to the average electric field at a certain brain location, $\vec{\boldsymbol{E}}$, can be written in a matrix form, $\vec{\boldsymbol{E}} = \overrightarrow{\boldsymbol{LF}} \cdot s$, where $\overrightarrow{\boldsymbol{LF}}$ is the leadfield. One way to write the objective function for least squares fitting of an unknown stimulation array, x, viz. arg minx$||\overrightarrow{\boldsymbol{LF}} \cdot x - \vec{\boldsymbol{E}}||^2$, is to minimize the L2-norm of the error, ($\overrightarrow{\boldsymbol{LF}}$.x-$\vec{\boldsymbol{b}}$), given a desired electric field distribution, $\vec{\boldsymbol{E}}$, e.g., to target the Purkinje cells of Crus

I/II in the fronto-cerebellar circuits (ROGERS et al., 2011). Here, it is important to limit the TMS generated electric field to the Purkinje cells in the Crus II to target for DLPFC executive network (Krienen and Buckner, 2009), which was achieved with cerebellar region-specific electric field modeling (Rezaee and Dutta, 2019) and neurophysiological validation (Batsikadze et al., 2019). Spill-over of TMS generated electric field to the dentate nuclei (DN) at higher TMS intensities will affect the recruitment curve (from inhibition to facilitation of the cerebellar-brain connection).

2.1 Supplementary Figures

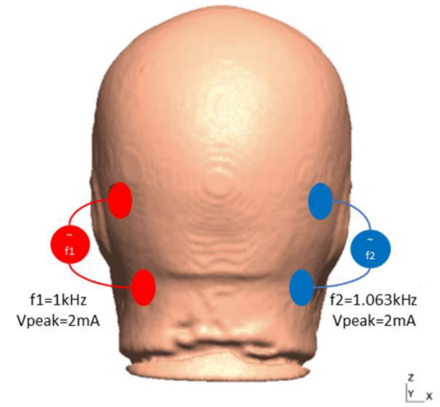

Supplementary Figure S1: An illustrative picture of transcranial temporal interference stimulation (tTIS) approach where two tACS sources with frequencies f1=1kHz and f2=1.063kHz are combined for amplitude modulation at 0.063kHz at the deep cerebellar nuclei (DCN).

At any region of the brain atlas, based on the leadfield (Rezaee and Dutta, 2019), the mean electric field ($\vec{E} = \overrightarrow{LF} \cdot s$) can be written as vector summation, $\vec{E} = \overrightarrow{LF\_PO9h} \cdot PO9h + \overrightarrow{LF\_PO10h} \cdot PO10h + \overrightarrow{LF\_Exx7} \cdot Exx7 + \overrightarrow{LF\_Exx8} \cdot Exx8$. Leadfield for the cerebellar lobules from the brain atlas (AAL (Rolls et al., 2020, 3), SUIT (Diedrichsen, 2006)) given below,

Cerebellar lobules:

1. 'Left I_IV'
2. 'Right I_IV'
3. 'Left V'
4. 'Right V'
5. 'Left VI'
6. 'Vermis VI'
7. 'Right VI'
8. 'Left Crus I'
9. 'Vermis Crus I'
10. 'Right Crus I'
11. 'Left Crus II'
12. 'Vermis Crus II'
13. 'Right Crus II'
14. 'Left VIIb'
15. 'Vermis VIIb'
16. 'Right VIIb'
17. 'Left VIIIa'
18. 'Vermis VIIIa'

19. 'Right VIIIa'
20. 'Left VIIIb'
21. 'Vermis VIIIb'
22. 'Right VIIIb'
23. 'Left IX'
24. 'Vermis IX'
25. 'Right IX'
26. 'Left X'
27. 'Vermis X'
28. 'Right X'
29. ‘Left Dentate Nucleus’
30. ‘Right Dentate Nucleus’

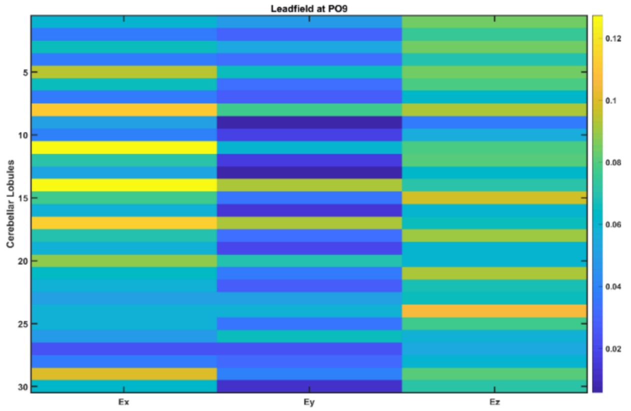

Supplementary Figure S2: Leadfield vector for PO9h at the 30 cerebellar lobules in the X, Y, and Z directions

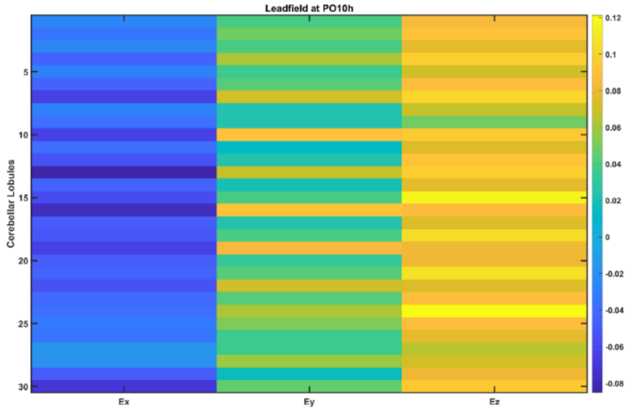

Supplementary Figure S3: Leadfield vector for PO10h at the 30 cerebellar lobules in the X, Y, and Z directions

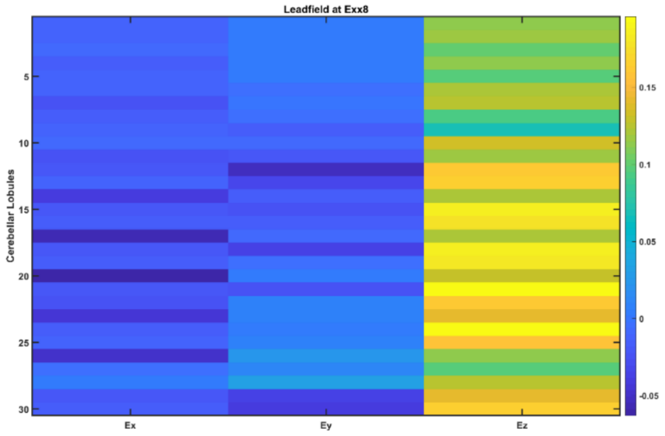

Supplementary Figure S4: Leadfield vector for Exx8 at the 30 cerebellar lobules in the X, Y, and Z directions

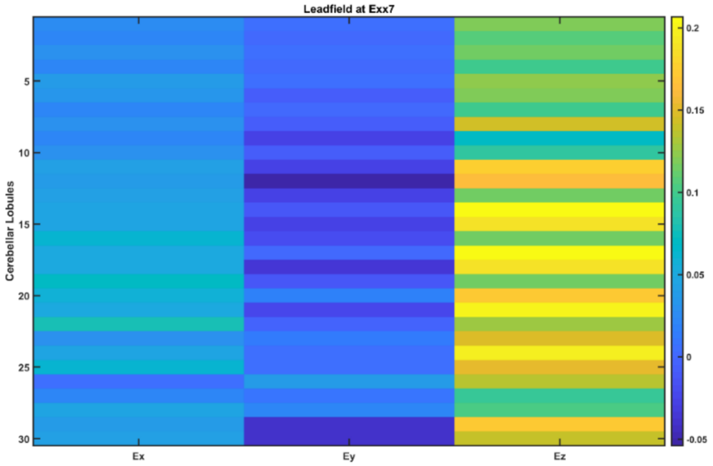

Supplementary Figure S5: Leadfield vector for Exx7 at the 30 cerebellar lobules in the X, Y, and Z directions

Circular electrodes placed at PO9h(-55.6, -70.1, -6.8) – Exx7(-54.6, -45.0, -54.0) location (shown in red in Figure S1) for tACS at 1kHz with 2mA as well PO10h(61.7, -69.7, -5.8) – Exx8(59.8, -49.3, -52.9) location (shown in blue in Figure S1) for tACS at 1.03kHz with 2mA in the X, Y, and Z directions (Figure S1). The superposition principle under Ohmic volume conductor (Leadfield approach) assumption was applied and the resultant mean electric field ($\overrightarrow{E} = \overrightarrow{LF} \cdot s$) at the cerebellar lobules are shown below.

3 Supplementary Tables and Figures: transcranial temporal interference stimulation of dentate nucleus

In our feasibility studies (Rezaee et al., 2021),(Rezaee et al., 2020), it was found challenging to optimize ctDCS electrode montage to solely stimulate the dentate nucleus without stimulating the lobes of the cerebellum (Abadi and Dutta, 2017). Therefore, we proposed this simulation study to leverage the neuronal sensitivity in transcranial temporal interference stimulation (tTIS) that depends on the neuronal membrane time-constant [84] for the dentate's focal stimulation nucleus. Here, tTIS can play
